# Units of Language Mixing: A Cross-Linguistic Perspective

**DOI:** 10.3389/fpsyg.2018.01719

**Published:** 2018-09-27

**Authors:** Artemis Alexiadou, Terje Lohndal

**Affiliations:** ^1^Department of English and American Studies, Humboldt University of Berlin, Berlin, Germany; ^2^Leibniz-Zentrum Allgemeine Sprachwissenschaft, Berlin, Germany; ^3^Department of Language and Literature, NTNU Norwegian University of Science and Technology, Trondheim, Norway; ^4^Department of Language and Culture, UiT The Arctic University of Norway, Tromsø, Norway

**Keywords:** English, German, Greek, Norwegian, Spanish, language mixing, distributed morphology

## Abstract

Language mixing is a ubiquitous phenomenon characterizing bilingual speakers. A frequent context where two languages are mixed is the word-internal level, demonstrating how tightly integrated the two grammars are in the mind of a speaker and how they adapt to each other. This raises the question of what the minimal unit of language mixing is, and whether or not this unit differs depending on what the languages are. Some scholars have argued that an uncategorized root serves as a unit, others argue that the unit needs to have been categorized prior to mixing. We will discuss the question of what the relevant unit for language mixing is by studying word-internal mixing in Cypriot Greek-English, English-Norwegian, Greek-English, Greek-German, and Spanish-German varieties that have been reported in the literature based on data from judgment experiments and spoken corpora. By understanding and modeling the units of language mixing across languages, we will gain insight into how languages adapt to each other word-internally, and what some possible outcomes of language contact are in the minds of speakers.

## Introduction

Much work on code-switching/code mixing/language mixing has aimed to provide a typology of possible mixing patterns across different languages. One example of this can be found in Muysken (2000, 2013) work. He defines mixing as lexical items and grammatical features from two (or more) languages that appear in one sentence. In Muysken (2000), he proposes a three-way typology which consists of insertion, alternation, and congruent lexicalization. Insertion involves insertion of well-defined chunks of language B into a sentence that otherwise belongs to language A. An example of this is provided in (1).

(1) Q’aya suya-wa-nki [*las cuatro*-ta]. (Quechua/Spanish)
  tomorrow wait-1OB-2SG at four-ACC
  Qo-yku-sqa-sun-ña [*bukis*]
  give-ASP-ASP-1PL-FUT-CON box
  ‘Tomorrow you wait for me at four. We’ll have a go at boxing.’ (Muysken, 2000, based on Urioste, 1966, p. 7)

In the case of alternation, two different languages A and B alternate within the same sentence, as shown in (2).

(2) a. maar’t hoeft niet *li-’anna ida šeft ana…*
  but it need not for-when I see I
  ‘but it need not be, for when I see, I…’ (Moroccan Arabic/Dutch; Muysken, 2000, based on Nortier, 1990, p. 213)
  b.Andale pues *and do come again*. (Spanish/English)
  ‘That’s all right then, and do come again.’ (Muysken, 2000, based on Gumperz and Hernández-Chavez, 1971, p. 118)

Congruent lexicalization is defined as the use of elements from either language in a structure that is wholly or partly shared by languages A and B. An example is provided in (3).

(3) Weet jij [**whaar**] Jenny is? (Dutch: Waar Jenny is)
  ‘Do you know where Jenny is?’ (English/Dutch; Crama and van Gelderen, 1984)

In Muysken (2013), a fourth type is added to the typology, namely backflagging. An example is given in (4).

(4) Q: What will you be when you grow up? (Dutch/Moroccan Arabic)
  A: Ik ben doctor *wella* ik ben ingenieur.
    I am doctor or I am engineer.
    ‘I will become a doctor or an engineer.’ (Nortier, 1990, p. 142)

Backflagging is defined as insertion of heritage language discourse marker in L2 discourse.

This typology is largely confined to word-level units and beyond. Recent work has begun to use language mixing as a probe into which basic units are put to use in both monolingual and bilingual speakers (González-Vilbazo and López, 2011; Alexiadou et al., 2015; Alexiadou, 2017a; Riksem et al., in press; Riksem, 2018). As we will see, these and other works argue that there are grammatical differences between varieties in terms of how mixing takes place. Our concern is precisely these differences rather than being able to predict exactly which mixing strategy a given speaker decides to use in a specific context; see Wohlgemuth (2009) for an extensive and typological investigation of the latter in the context of mixing involving verbs.

The goal of the present paper is to synthesize and compare the current findings from various bilingual populations, in particular heritage language speakers. We will focus on word-internal mixing in Cypriot Greek-English, English-Norwegian, Greek-English, Greek-German, and Spanish-German varieties based on data from judgment experiments and spoken corpora.

This paper is organized as follows. The section “Background” provides some background, in particular concerning the nature of word-internal language mixing. Then we move on to the case studies reviewed in the paper. The section “Word-Internal Mixing in Varieties Involving Greek” will consider word-internal mixing in Greek-German and Cypriot Greek-English, whereas the section “Word-Internal Mixing in German-Spanish” will look into Spanish-German. The section “Word-Internal Mixing in Varieties Involving Norwegian” is devoted to word-internal mixing in varieties involving English and Norwegian. In the section “Word-Internal Mixing in Telugu,” we zoom out and consider an entirely different variety typologically speaking, namely Telugu. The section “Discussion and Analysis” will discuss and compare the patterns across the different varieties and also comment on recent work by López (2018). Lastly, the section “Conclusion” concludes the paper.

## Background

This section will provide some context and relevant background for the present paper. In the Section “The Nature of Word-Internal Mixing,” we discuss the notion of word-internal mixing, situating it within a long research history in work on language mixing. We then also discuss why word-internal mixing is important for modeling multilingual’s linguistic competence.

### The Nature of Word-Internal Mixing

Since Poplack (1980), there has been significant work on the issue of word-internal language mixing, or code-switching as most of the relevant literature labels it. Her work can in many ways be seen as one of the initiators to what MacSwan (2014, p. 4) calls the ‘constraint-based research program.’ She proposed two constraints, given in (5) and (6).

(5) The Equivalence Constraint
  Codes will tend to be switched at points where the surface structures of the languages map onto each other.

(6) The Free Morpheme Constraint
  A switch may occur at any point in the discourse at which it is possible to make a surface constituent cut and still retain a free morpheme.

We won’t discuss the first constraint (5), but the second constraint is quite important for the present paper. Sankoff and Poplack (1981) developed (6) further and stated it as follows:

(6) The Free Morpheme Constraint Revisited
  A switch may not occur between a bound morpheme and a lexical form unless the latter has been phonologically integrated into the language of the bound morpheme.

The formulation in (6) does not allow examples like in (7), but it allows examples like (8).

(7) a. ^∗^**eat**-iendo
  eat-ing
  ‘eating’ (Poplack, 1980, p. 586)
  b. **run**-eando
  run-ing
  ‘running’ (Sankoff and Poplack, 1981, p. 5)

(8) a. **flip**-eando
  flip-ing
  ‘flipping’ (Sankoff and Poplack, 1981, p. 5)
  b. **parqu**-eando
  park-ing
  ‘parking’ (MacSwan, 2005, p. 7)

The reason is that *run* is has a clear English phonology with a mid-central vowel [∧] which is not part of Spanish phonology. In (8), on the other hand, *flip* and *parqu* have been accommodated to Spanish phonology. Since then, the distinction between (7) and (8) has often been referred to as one of code switching vs. borrowing. A borrowed word is phonologically integrated into the recipient language, whereas code switches are taken from two different lexicons.

In the language contact literature, there are two distinct positions concerning switching and borrowing. The first position sees code switching and borrowing as part of a continuum (e.g., Myers-Scotton, 1993, 2002, 2006; van Coetsem, 2000; Thomason, 2003). The second position rather views code switching and borrowing as two distinct processes (Sankoff and Poplack, 1981; MacSwan, 1999; Poplack and Dion, 2012; MacSwan and Colina, 2014; Poplack, 2018). In a thorough review of the debate, Grimstad (2009, pp. 6–7), based on Matras (2009, p. 106) and Muysken (2000, p. 69), characterizes the two positions as in (9).

(9) a. Borrowing is the diachronic process by which languages enhance their vocabulary (or other domains of structure), while code-switching is instances of spontaneous language mixing in the conversation of bilinguals. Borrowed items originate as code-switches.
  b. Code-switching involves inserting alien words or constituents into a clause; borrowing involves entering alien elements into a lexicon.

However, as Grimstad also points out, when dealing with these notions, it is worth keeping the following quote from Gardner-Chloros (2009, pp. 10–11) in mind:

Code-switching (CS) is not an entity which exists out there in the objective world, but a construct which linguists have developed to help them describe their data. It is therefore pointless to argue about what CS is, because, to paraphrase Humpty Dumpty, the word CS can mean whatever we want it to mean.

As is to be expected given this situation, the asymmetry between (7) and (8) is controversial in the literature. Several scholars have argued for it (e.g., Bentahila and Davies, 1983; Berk-Seligson, 1986; Clyne, 1987; MacSwan, 1999), whereas others have presented counterexamples (e.g., Nartey, 1982; Bokamba, 1989; Myers-Scotton, 1993; Halmari, 1997; Chan, 1999; Hlavac, 2003; Grimstad et al., 2014, 2018; Grimstad, 2017; Riksem, 2018). MacSwan and Colina (2014, p. 203) note that ‘[i]n some cases, researchers have not adequately documented the phonological characteristics of items to permit us to judge their level of integration, and in others assumptions about the morphological status of elements have not been made explicit.’ This suggests that mixing or switching is primarily a phonological phenomenon:

In an important respect, then, language switching *is* phonological switching. When we stop speaking one language and begin speaking another, the shift is prominently characterized by a change in the way we say words. So conceived, the relevant research question would appear to revolve around discovering the conditions under which one can switch from one phonological system to another (MacSwan and Colina, 2014, p. 188).

MacSwan proposes the PF Interface Condition as a way of modeling this claim. The condition is given in (10) (MacSwan, 2009, p. 331; MacSwan and Colina, 2014, p. 19).

(10) PF Interface Condition
  (i) Phonological input is mapped to the output in one step with no intermediate representations.
  (ii) Each set of internally ranked constraints is a constraint dominance hierarchy, and a language-particular phonology is a set of constraint dominance hierarchies.
  (iii) Bilinguals have a separately encapsulated phonological system for each language in their repertoire in order to avoid ranking paradoxes, which result from the availability of distinct constraint dominance hierarchies with conflicting priorities.
  (iv) Every syntactic head must be phonologically parsed at Spell Out. Therefore, the boundary between heads (words) represents the minimal opportunity for code-switching.

(10) builds on the PF Disjunction Theorem in MacSwan (1999, 2000). MacSwan (1999, 2009,2013) further argues that mixing in contexts of head movement is prohibited, which (10iv) derives given that a word is defined as ‘a lexical head (X^0^) whose morphological composition has been determined internally within the lexicon’ (MacSwan, 2005, p. 11).

However, there are several issues with MacSwan’s and others’ approach in terms of banning word-internal mixing. Here, we will highlight two.

To begin with, a phonological definition is problematic if not simply because words are hard to define simply based on phonological criteria, as the following quote by Jespersen (1924/1963, pp. 92–93) makes clear:^1^

Words are linguistic units, but they are not phonetic units: no merely phonetic analysis of a string of spoken sounds can reveal to us the number of words it is made up of, or the division between word and word. […] As, consequently, neither sound nor meaning in itself shows us what is one word and what is more than one word, we must look out for grammatical (syntactic) criteria to decide the question.

Poplack (2013, p. 12), in a discussion of what we have learned from Poplack (1980) till today also highlights the following: ‘Phonological and morphosyntactic integration are independent. Phonology of both CS and B, is variable, and thus cannot reliably be used to distinguish between them.’ Again, relying on phonological criteria alone is problematic.

The second reason is related to the concept of the lexicon that is assumed (see also Grimstad, 2017). As (9) makes clear, the distinction between borrowing and mixing generally invokes the question of whether a given unit is part of the lexicon of the recipient language or the donor language. This assumes, as in e.g., Muysken (2000) model, that a multilingual speaker has an individual mental lexicon for every language she knows. However, a contemporary speaker has no access as such to information about which lexicon a particular element belongs to or how it became a member of that lexicon. MacSwan (2005, p. 11) makes this point clear in the context of distinguishing between borrowing and borrowing for the nonce:^2^

For the purposes of a synchronic theory of language contact, the distinction between BORROWING and NONCE BORROWING is unimportant: The difference in meaning depends on a word’s history – inaccessible to a linguistic system represented in the mind/brain of an individual.

The same argument could be made for mixing more generally. In the present paper, we will make such an argument as part of developing a non-lexicalist approach to language mixing (cf. González-Vilbazo and López, 2011; Pierantozzi, 2012; Bandi-Rao and den Dikken, 2014; Alexiadou et al., 2015; Grimstad et al., 2014, 2018; Merchant, 2015; Lillo-Martin et al., 2016; Alexiadou, 2017b; Grimstad, 2017; Riksem, 2017, 2018). This work tries to respond to the following challenge posed by MacSwan (2013, 2014, pp. 347: 18): ‘Whether a sufficiently rich non-lexicalist theory involving late insertion, such as distributed morphology […], could achieve similar results [to lexicalist approaches] has not been investigated.’ An explicit such model has been provided in López (2018), the aim being to develop a minimalist Distributed Morphology model of code-switching, labeled MDM in his work. From an MDM perspective, bilinguals have only one list containing the roots from their two languages, List 1 in Distributed Morphology, and only one list containing the vocabulary insertion rules of their two languages, List 2 in Distributed Morphology. Put differently, multilinguals have more vocabulary items at their disposal to realize a particular syntactic structure. We will come back to this, in particular in the section “Discussion and Analysis.”

The current contribution is intended to further the work done in the emergent non-lexicalist program, in particular by contributing a larger cross-linguistic picture of patterns of word-internal mixing.

### Multilingual Individuals and Their Linguistic Competence

For a long time, formal approaches to grammar have explicitly or implicitly followed the idealization set forth in Chomsky (1965, p. 3):

Linguistic theory is concerned primarily with an ideal speaker-listener, in a completely homogeneous speech-community, who knows its language perfectly and is unaffected by such grammatically irrelevant conditions as memory limitations, distractions, shifts of attention and interest, and errors (random or characteristic) in applying his knowledge of the language in actual performance.

This idealization also relates to the distinction between competence and performance outlined in Chomsky (1965). Competence is the tacit linguistic knowledge a speaker has, whereas performance is the employment of this knowledge in actual production. In formal grammar, the focus has been on developing competence models based on the linguistic performance of speakers.

This has been a successful research strategy insofar as it has uncovered a range of new generalizations and theoretical proposals concerning the unique human ability for language. However, if the models are going to be ecologically valid, they clearly need to generalize beyond monolinguals. Chomsky (1986) emphasizes the notion of I-language, which connotes an individual, internal, and intensional language. A core task has been to try to answer the question of what a possible mental grammar is. The hypothesis is that there are constraints on what can be a human mental grammar, constraints that may or may not hold cross-linguistically. The theories and models that are developed should simultaneously include the possible structure and exclude the impossible ones.

From this perspective, it is obvious why studying multilingual individuals is crucial if you want to discover the potential range of human grammars. The current contribution focuses on word-internal language mixing, which is but one of many aspects of multilinguals’ knowledge and use of language.

Within the literature studying language mixing, it is either argued that special mechanisms are needed to account for language mixing or that such mechanisms are not needed. Null theories or constraint-free theories are theories of the latter type, they assume that mixing is not constrained by special rules unique to mixing (cf. Mahootian, 1993; MacSwan, 1999, 2000, 2005, 2014; González-Vilbazo and López, 2011, 2012; Pierantozzi, 2012; Bandi-Rao and den Dikken, 2014; Grimstad et al., 2014, 2018 Alexiadou, 2017a). Rather, ‘exactly the same principles which apply to monolingual speech apply to code-switching’ (Mahootian, 1993, p. 3). This aligns with the following quote from Muysken (2000, p. 3):

The challenge is to account for the patterns found in terms of general properties of the grammar. Notice that only in this way can the phenomena of code-mixing help refine our perspective on general grammatical theory. If there were a special and separate theory of code-mixing, it might well be less relevant to general theoretical concerns.

In what follows, we will adopt this perspective and make use of it in our comparative analysis of word-internal language mixing.

## Word-Internal Mixing in Varieties Involving Greek

In this section, we will consider word-internal mixing in languages that are mixed with Greek, notably English and German. The section “The Verbal Domain” discusses the verbal domain whereas the section “The Nominal Domain” is concerned with the nominal domain. Our goal is not to develop and motivate previous in-depth analyses of the data, rather to present the data and the gist of the analysis for the sake of the cross-linguistic comparison in the section “Discussion and Analysis.”

### The Verbal Domain

Alexiadou (2017a) discusses word internal mixing in two Greek varieties, English-Cypriot Greek, and German-Greek. Both these varieties make use of two different patterns when it comes to mixing: What is typically labeled the light verb construction (LVC) pattern, and the affixal pattern. These are illustrated in (11) for Greek-German.

(11) a. kano **abschalten**
  do.1SG kick.back.INF
  ‘I am kicking back.’ (Alexiadou, 2011: ex. [12])
  b. **skan**-ar-o
  scan-AFF-1SG
  ‘I am scanning.’ (Alexiadou, 2011: ex. [13])

Alexiadou (2017a, p. 174) provides further details about the sociolinguistic context of these data; see her paper for further discussion and references. She also proposes an analysis of the LVC pattern, a pattern that we won’t focus on in the current paper.

As shown in (12), the affixal pattern also exists in the Cypriot Greek-English variety.

(12) a. **muv**-ar-o
  move-AFF-1SG
  ‘I am moving.’
  b. **kansel**-ar-o
  cancel-AFF-1SG
  ‘I am canceling.’ (Gardner-Chloros, 2009, pp. 50–51)

In these examples, we see that the Greek affix attaches to the German and/or English root. A dedicated affix, -*ar*-, is used to verbalize the root. This particular affix triggers stress shift to the penultimate syllable. Even though it is used less frequently than many other verbalizing affixes in Modern Greek, it is the default verbalizer in these mixing varieties.

Alexiadou (2011) observes that the affixal patterns are not in free distribution in Greek-German. (11a) and (12b) contain a monosyllabic root, yet not all monosyllabic roots can take part in the affixal pattern. As an example, (13) contains a monosyllabic root, yet only the light verb strategy is licit.

(13) na kanun kämpfen
  SUBJ do.3PL fight.INF
  ‘They should fight.’ (Alexiadou, 2011: ex. [25])

Furthermore, it is always an English/German root which combines with a Greek affix. The combination of a Greek root with a German inflection is rejected by speakers.

We will now consider how Alexiadou (2017a) accounts for the asymmetries. Her analysis is based on Distributed Morphology (Halle and Marantz, 1993; Embick and Noyer, 2007; Embick, 2015), where syntactic categorization takes place in the syntax via the presence of functional heads. Roots are category-neutral and need to be categorized in the syntax by way of merging with a functional head (see Alexiadou and Lohndal, 2017b for more on various analytical possibilities). Little v turns a root into a verb whereas little n turns a root into a noun, as shown in (14).

**Table:** 

(14) a.	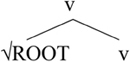	b.	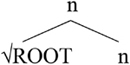

More concretely, for a string like (15a), from Embick (2004, p. 365), the structure is provided in (15b).

**Table:** 

(15)	a.	The metal flatt-en-ed.
	b.	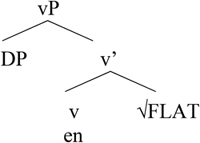

In languages like Greek, there are many verbalizing affixes (see Alexiadou, 2017a, p. 179 and references therein). These are typically taken to realize little v.

Alexiadou (2017b, p. 182) proposes the following structure for the affixal pattern.

**Table:** 

(16)	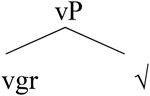

In Greek, there is a kind of default affix that is used to verbalize non-native roots. This affix can be used in a range of contexts. Put differently, this default affix (v) can be seen as incorporating into a non-native root, cf. Bandi-Rao and den Dikken (2014).

### The Nominal Domain

Alexiadou (2011, 2017b) and Alexiadou et al. (2015) discuss cases of word internal mixing in the noun phrase involving Greek-German and Greek-English pairs. All the examples provided are of the following form: the involve a German or English stem to which a Greek affix is added.

**Table:** 

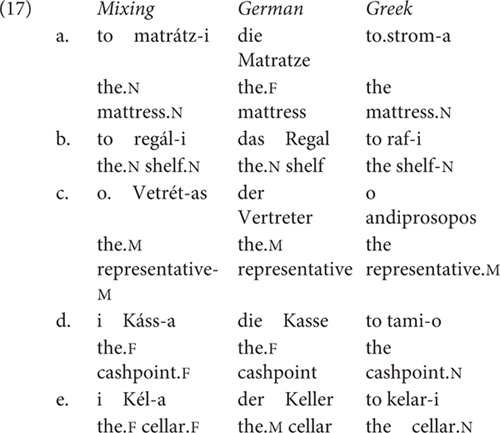

Gardner-Chloros (2009, p. 50) reports the following Greek-English mixing cases in the variety of British born Cypriot Greek speakers:

**Table:** 

18		*BBC Mixing*	*English*	*Greek*
	a.	marketa (F)	market	agora (F)
	b.	hoteli (N)	hotel	ksenodohio (N)
	c.	kuka (F)	cooker	furnos (M)
	d.	fishiatiko (N)	fish and chip shop	–
	e.	kitsi (N)	kitchen	kuzina (F)
	f.	ketlos (M)	kettle	–
	g.	haspas (M)	husband	andras (M)


Fotopoulou (2004) cites Greek-German examples where no inflection is added to the German noun and the determiner comes from Greek:

**Table:** 

(19)	mu pire tin	Ausfahrt
	me took the.F.ACC exit	

Goula (2017) observes similar cases of mixing in Greek-English, which she tested experimentally. When the morphology of the noun is not adapted, the determiner may come from Greek. She moreover notes that sometimes the determiner bears default gender, e.g., neuter for inanimates, see Tsimpli and Hulk (2013) and Anagnostopoulou (2017b) for recent discussion, or carries over the gender of its Greek translation equivalent, masculine in the example below. This the so-called analogical gender strategy, see López (2018) for further discussion.

**Table:** 

(20)	a.	to map	b.	o map
		the.N map		the.M map

Goula (2017) shows that the translation equivalence choice was preferred in the comprehension task, while the default choice was preferred in the production task.

Alexiadou (2011, 2017b) proposes the structure in (21): gender and inflection class information are on n. In fact, the nominal mixing data support the view that neither gender is a property of roots, as argued for in detail in Kramer (2015) nor inflection class, as they can be freely assigned structurally.

**Table:** 

(21)	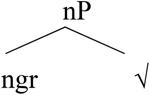	word-internal

## Word-Internal Mixing in German-Spanish

In this section, we will consider word-internal mixing in a variety whereby German and Spanish are mixed. Before we turn to the verbal and nominal domains, a brief note about the data is in order. The data are taken from González-Vilbazo (2005) and González-Vilbazo and López (2011). They report that the data come from the German School of Barcelona. This school consists of between 1,000 and 1,400 students who from an early age generally have a high exposure to both languages. Their informants belong to a homogenous socio-economic community whereby the majority are Spanish/German bilinguals. This multilingual environment, much like other multilingual environments, make use of language mixing. As González-Vilbazo and López (2011, p. 837) report, the students are proud of their mixing practices.

### The Verbal Domain

González-Vilbazo and López (2011) show that mixing happens word-internally, as depicted in (22).

**Table:** 

(22)	Wir	*utilis*ieren	spanische	Wörter,	die
	We	use	Spanish	words	than
	dann	**alemanis**iert	werden	**y**	
	then	Germanized	are	and	
	**hacen**	klingen	**un**	**poco**	**raro**
	do	sound	a	bit	strange


‘We use Spanish words, that are then Germanized and sound a bit strange.’

(González-Vilbazo and López, 2011, p. 833)

In word internal mixing, speakers are able to combine a Spanish root with a German verbal inflection, as shown in (23). However, speakers cannot in general combine a German root with a Spanish infinitival inflection (24) (González-Vilbazo and López, 2011, p. 835).

**Table:** 

(23)	a.	**cos**-ier-en	b.	**utilis**-ieren
		‘sew’		‘use’
(24)	a.	^∗^benutz-**ear**	b.	^∗^näh-**ear**
		‘use’		‘sew’

As González-Vilbazo and López (2011, p. 835) emphasize, in the German-Spanish variety “German/Spanish bilinguals accept (and produce) nonce words created by joining together a Spanish root and a German verbal inflection. However, these same bilinguals reject a word made up of a German root and a Spanish verbal inflection.” As we saw above, the reverse is observed in mixing varieties of Greek: A German or English root always combines with a Greek affix, and the combination of a Greek root with a German inflection is rejected by speakers.

According to González-Vilbazo and López (2011), every Spanish verb carries a specification for its conjugation class. Furthermore, v bears unvalued features for conjugation class. In order to value this feature, V-to-v movement needs to take place. Crucially, German verbs do not carry a specification for conjugation class. Therefore, it cannot be the case that a German verbal root could incorporate into a v that is specified for conjugation class. However, a Spanish verbal root can be embedded and incorporate into a German v because this v is unspecified for conjugation class, as in (25b). The light v is always realized with the Spanish verb in (25a).

**Table:** 

(25)	a.	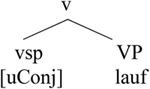
	b.	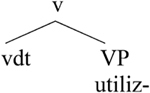

### The Nominal Domain

González-Vilbazo (2005, p. 141 f.) notes that nominal word internal mixing is less frequent in the Spanish-German variety, but it appears to obey a morphological restriction similar to that of verbal mixing: a Spanish affix cannot attach to a German stem, while the reverse is allowed:

(26) a. ^∗^**Stuhl-**o
  chair.MASC
  b. **Segurat-**en
  security.man-PL

González-Vilbazo (2005) notes that such nouns end in -*e* in the singular, while they take the affix -*en* in the plural. The singular marking suggests that they are not Spanish nouns as they should end in -*a*. He argues that this case is different from that of verbal mixing: In the verbal mixing there is overt verbalizing morphology, e.g., *ier*- that enables the further suffixation of German inflectional material. This affix creates a German verbal stem to which further German affixes can be added. This is not the case in the nominal domain. The Greek mixing data seen in the previous section further support this. There is no overt nominalizing morphology present. To this end González-Vilbazo (2005) suggests that a covert affix is present that creates a German base to which further affixation is possible. We note that within Distributed Morphology, this intermediate step is not necessary: Little n is the nominalizer and carries all inflection. From this perspective, in Spanish-German the direction of affixation is as shown in (27).

(27) Spanish root + German affix

This is the reverse in the Greek-German/English cases:

(28) German/English root + Greek affix

González-Vilbazo (2005) further cites examples which do not involve affixation, but as we have seen above for Greek, a determiner from Spanish in combination with a German noun. Put differently, Spanish functional material is able to combine with a German root.

**Table:** 

(29)	a.	la Stunde	b.	el Lehrer
		the hour		the teacher
		‘the hour’		‘the teacher’

In (29), the gender of the article corresponds to the gender of the German noun. As Spanish lacks neuter, all German nouns that are neuter are preceded by the Spanish masculine article, which is the default gender in the language.

The reverse pattern is also found:

(30) die ley
  the.F law
  ‘the law’

This latter case is more complex. As González-Vilbazo (2005) details, feminine Spanish nouns are preceded by the feminine German determiner no matter the gender of the German translation equivalent (in this case, neuter). In other words, this group preserves its gender. In the case of masculine Spanish nouns, the only articles that are allowed are those that are syncretic for masculine and neuter. As a result, indefinite determiners are preferred, as shown in (31a). The only exception discussed involves genitive case, where no switch is allowed, although both masculine and neuter indefinite articles bear the same form, (31b). According to López (2018), this is so as German nouns often bear genitive morphology themselves, i.e., there is a concord effect between the determiner and the noun, (31c). As Spanish lacks case morphology, genitive German inflection is blocked from appearing on a Spanish noun. This contrast suggests to us that not only gender but also case inflection is on n, as argued for Greek in Alexiadou (2017b), and see Anagnostopoulou (2017a) for a general claim on the relationship between n and case. Since n is Spanish, no case morphology can appear there and concord is blocked:

**Table:** 

(31)	(Spanish = masculine, German = neuter)		
	a.	?der/ein	cuaderno
		DEF/INDEF	notebook	
	b.	^∗^eines	tenedor	
		INDEF.GEN	fork	
	c.	eines	Mannes	
		INDEF.GEN	man.GEN	

In the next section, we turn to a different pair of languages, namely English and Norwegian.

## Word-Internal Mixing in Varieties Involving Norwegian

In this section, we will consider mixing of varieties where Norwegian is one of the languages. We will consider two varieties: the heritage language American Norwegian in the section “Mixing in American Norwegian,” and then Norwegians in Norway who mix English into their Norwegian in the section “Mixing of English and Norwegian in Spoken Norwegian.” As we will see, the same patterns are found in both varieties.

### Mixing in American Norwegian

American Norwegian is a heritage language of Norwegian. It is spoken in North America, mainly in the United States. Its speakers today are descendants of immigrants who came from Norway approximately from the 1850s until the 1920s. This makes American Norwegian a minority language which exists in a language community significantly dominated by English. All American Norwegian speakers share the following characteristics: American Norwegian is their L1, and in many cases this was their only language up until school age. In recent decades, all speakers of American Norwegian have been heavily English-dominant, resulting in significant lexical access issues when speaking American Norwegian. This means that they often display a mixture of the two languages, making their speech ideal for studying language mixing (Grimstad, 2018; Riksem, 2018).

Haugen (1953) conducted the first large-scale investigation of American Norwegian. He provides examples like the following.

(32) Så **play**-de dom **game**-r
  then play-PAST they game-PL
  ‘Then, they played games’

(33) Så **happ[e]n**-a de så at e kåm inn på **office**-en te
  so happen-PAST it so that I came in to office-DEF to
  statskasserar-en då
  national.treasurer-DEF then
  ‘So it happened that I came into the office of the national treasurer.’

More recently, the establishment of the *Corpus of American Nordic Speech* (Johannessen, 2015) has generated a lot of new work on American Norwegian (see e.g., the summary in Riksem, 2018). In particular, Grimstad et al. (2014), Riksem (2018), Riksem et al. (in press), and Grimstad et al. (2018) have studied language mixing based on corpus data from 50 speakers of American Norwegian. These speakers are all between 70 and 100 years of age and constitute probably the last generation of American Norwegian speakers. The following discussion will be based on data from Riksem et al. (2017).

In general, Norwegian is the main language while the other is the secondary language (Åfarli, 2015). The main language can be argued to provide the overall grammatical structure, including more or less all derivational and inflectional morphology. In many cases, the lexical items also come from the main language, but when they do not, they come from the secondary language. Åfarli, 2015 and Riksem et al. (2018) depict this as in (34) where L stands for lexical item and INFL for inflectional morphology.

(34) a. L_SEC_ + INFL_MAIN_
  b. L_MAIN_ + INFL_MAIN_
  c. ^∗^L_SEC_ + INFL_SEC_
  d. ^∗^L_MAIN_ + INFL_SEC_

(34c) does not hold for bigger mixed chunks, and some other cases studied by Grimstad (2017); see her work for details.^3^

Riksem et al. (in press) provide a range of examples of verb-internal and noun-internal mixing. The former is illustrated in (35) and the latter in (36).

**Table:** 

(35)	a.	**spend**-e	b.	**rais[e]**-er	c.	**catch**-a
		*spend*-INF		*raise*-PRES		*catch*-PAST
		‘to spend’		‘raise(s)’		‘caught’
		(blair_		(blair_WI_		(sunburg_
		WI_02gm)		01gm)		MN_03gm)
(36)	a.	**road**-en	b.	**voting**-a	c.	**fenc[e]-**a
		*road*-		*voting*-		*fenc[e]-*
		DEF.SG.M		DEF.SG.F		DEF.PL.N
		‘the road’		‘the voting’		‘the fences’
		(webster_		(westby_		(coon_valley_
		SD_02gm)		WI_01gm)		WI_06gm)

In the verbal cases, we see that an English item can acquire both the infinitival form, the present tense and the past tense (see Eide and Hjelde, 2015 for more on tense in American Norwegian). In the nominal forms, the nouns can be inflected according to definiteness, number, and gender/declension class.

Riksem et al. (in press) analyze the mixing cases in (35) and (36) as cases whereby an English root is embedded into a Norwegian grammatical structure. Crucially, the English roots do not have any grammatical features. Rather, features are merged in the functional spine and morphophonological exponents come to realize them. An abstract structure for the American Norwegian noun phrase can be illustrated in (37) (Riksem et al., in press).

**Table:** 

(37)	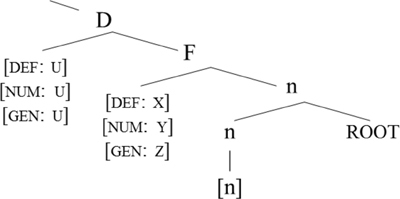

In this structure, definiteness, number, and gender are all encoded on *one* functional projection. It could also be that gender is encoded on n (Alexiadou, 2004, 2017a; Kramer, 2015), this particular choice does not matter for present purposes. The features then combine with the root to yield the actual exponent, as shown in (38).

**Table:** 

(38)	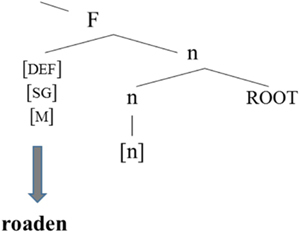

A similar logic underlies verbal mixing. An abstract structure is provided in (39), and here, the root again combines with the tense morpheme to yield the exponent *renter* ‘rents.’ Again, the structure is taken from Riksem et al. (in press); see that paper for additional justification of this particular structure.

**Table:** 

(39)	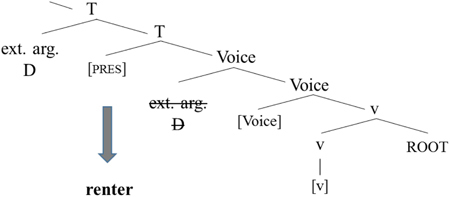

In general, then, we see that English roots can combine with Norwegian functional material to yield instances of word-internal mixing.

### Mixing of English and Norwegian in Spoken Norwegian

Norwegians generally have a high proficiency in English, in particular the younger generations. In Norway, it is well-known that they often mix English words into their Norwegian. A recent study by Sunde (2016) investigates a gaming community, which is a community where English is especially important. Based on oral and spoken data, Sunde (2016) argues that Norwegian is clearly the matrix language in Myers-Scotton (1993) sense, as it is the language contributing the morphosyntactic frame. That is, Norwegian determines the order of morphemes and functional morphology. This corresponds to what we in the section “The Verbal Domain” called main language, which is a more general label not specifically associated with Myers-Scotton’s implementation. One example of this is provided in (40); the translation into English is ours (Sunde, 2016, p. 138).

(40) Selv om han **trad**a seg sjøl ut […] så lot han fortsatt
  even if he traded REFL self out so let he still
  **team mat**en sin som
  team mate his who
  var på A værende igjen aleine, og han blei
  was on A remaining again alone and he was
  **overwhelm**a av alle terroristene […]
  overwhelemed by all terrorists.DEF
  ‘Even if he traded himself out, he still let his team mate, who remained on A, behind, and he was overwhelmed by all the terrorists.’

Sunde (2016, p. 140) shows that instances of infinitival, present and past tense forms occur. Some of her examples are given in (41).

(41) a. De har ikke tid til å **defus**e bomben.
  they have not time to to defuse bomb.DEF
  ‘They do not have time to defuse the bomb.’
  b. **Carry**er deg lett ut.
  carry you easily out
  ‘I easily carry you out.’
  c. Nå **overextend**a de veldig.
  now overextended they a.lot
  d. Vet at jeg har **leav**et før.
  know that I have left before
  ‘I know that I have left before.’

Turning to word-internal mixing in the nominal domain, Sunde (2016, p. 143) provides examples such as (42).

(42) a. Fant **trad**en
  found trade.DEF
  ‘I found the trade.’
  b. Jeg kommer til å holde den **scout**en
  I come to to holde that scout.DEF
  ‘I will hold that scout.’
  c. **Inspect** kniven i **inventory**en min
  inspect knife.DEF in inventory.DEF my
  ‘Inspect the knife in my inventory.’

Again, we see that the lexical items can come from English whereas the morphology comes from Norwegian.

The same analysis as Riksem et al. (in press) develop for American Norwegian can also be used for the data seen in this sub-section: English roots are merged into structures based on Norwegian features. No further assumptions need to be made.

## Word-Internal Mixing in Telugu

In this section, we will consider data from Classical Telugu (a South-Central Dravidian language) reported by Bandi-Rao and den Dikken (2014). The data are based on acceptability judgments. They observe an asymmetry similar to the one we have observed for other pairs discussed above when looking at a mixing variety of English-Telugu: Only Telugu roots can combine with English -*ify*. It is not possible for an English root to combine with the Telugu -*inc* affix, as the contrast between (43) and (44) shows.

**Table:** 

(43)	a.	My sister **kal(i)p**-ified the curry.	*kalp* ‘stir’
	b.	You have to **kar(i)g**-ify the butter	karg ‘melt’
	c.	The teacher made the child	**Ed(i)c**-ify in school. *Edc* ‘cry’
		(Bandi-Rao and den Dikken, 2014, p. 163)

(44) ^∗^*vaaDu nanni love-inc-EEDu*.
  he.NOM me.ACC love-DO-PST.AGR
  ‘He loved me.’ (Bandi-Rao and den Dikken, 2014, p.165)

The authors attribute this to the fact that Telugu affix is an incorporator, while the English affix is not. They relate this to English systematically disallowing incorporation into verbal heads. For example, English does not allow (45) but instead makes use of (46).

(45) a. ^∗^John meat-eats.
  b. ^∗^John up-looked the number.

(46) a. John eats meat.
  b. John looked up the number.

If incorporation were to take place in (44), an ill-formed head at PF would be the result, assuming that mixing below the head-level is banned (MacSwan, 1997, 2000), a claim Bandi-Rao and den Dikken (2014) endorse. Thus, it is avoided. By contrast, the Telugu root and the English affix only come together as a unit at PF, i.e., the Telugu root never incorporates into -*ify* because principles of English grammar do not license it.

## Discussion and Analysis

The sections “Word-Internal Mixing in Varieties Involving Greek,” “Word-Internal Mixing in German-Spanish,” “Word-Internal Mixing in Varieties Involving Norwegian,” and “Word-Internal Mixing In Telugu” demonstrate that there are some interesting differences between the various varieties. As González-Vilbazo and López (2011, p. 835) emphasize, mixing between a Spanish root and a German verbal inflection is fine, but the same individuals reject an element consisting of a German root and a Spanish verbal inflection. Alexiadou (2017a, p. 167) notes that the asymmetry is the reverse for Greek mixing varieties: It is always a German/English root combining with a Greek affix. For American Norwegian, the root can be either Norwegian or English, but we generally do not find a Norwegian root with English inflection (Grimstad, 2017; Grimstad et al., 2018). This is also the case in the nominal domain. Furthermore, Telugu displays an asymmetric pattern whereby Telugu roots can combine with English functional morphology but English roots cannot appear together with Telugu functional morphology. The following table offers an overview of the patterns seen in our survey.

The variation displayed in **Table 1** raises the question of what the source behind these various asymmetries are.

**Table 1:** Possible and impossible patterns of word-internal mixing.

Language pair	Possible mixing		Impossible mixing	
		
	root	inflection	root	inflection
German-Spanish	Spanish	German	German	Spanish
German-Greek	German	Greek	Greek	German
English-Greek	English	Greek	Greek	English
English-Norwegian	English	Norwegian	Norwegian	English
English-Telugu	Telugu	English	English	Telugu


One potential answer to this question is to suggest that the asymmetries we observe are simply an effect of the main language. In other words, the morphosyntactic spine comes from the language whose affixes the speakers employ, i.e., Greek, English, Norwegian and German, respectively (cf. also Grosjean, 2008, 2013 on the notion of ‘language mode’). However, it is important to clarify what we mean by main language. For instance, Myers-Scotton (1993, 2002) and Jake et al. (2002) argue that language mixing necessitates a distinction between matrix language and embedded language. A matrix language is the main language of the speaker and it has a grammatical correlate: It is responsible for word order and for providing functional morphemes. The embedded language can provide lexical items. Scholars have extensively discussed the predictions and factual accuracy of the matrix language model (see MacSwan, 2014, pp. 14–16 and references therein). We follow Åfarli (2015) and Riksem et al. (in press) in arguing that main and secondary languages, to the extent that they are valid, are observational phenomena, not theoretical primitives.

Evidence that this may be problematic as a general answer is provided by the Telugu cases since there, it is the secondary language that provides the functional morphology. Furthermore, as González-Vilbazo (2005) observes, speakers reject forms where a Spanish affix attaches to a German stem. Such data suggest that the relevant factor cannot be the division of labor between main language and secondary language. Importantly, though, a caveat is in order. The crucial data from both Telugu and German-Spanish are based on acceptability judgments (see Toribio, 2001a,b on the latter in studying mixing). In other work on language mixing, it has been observed that judgments are not always reliable indicators of the underlying grammar. Let us consider one such example.

In mixing between English and Spanish, Moro (2014) reports that whereas speakers accept the pattern in (47), they reject the pattern in (48).

(47) a. el **employer**
  ‘the employer’
  b. la **washing machine**
  ‘the washing machine’

(48) a. ^∗^**the** casa
  ‘the house’
  b. ^∗^**the** vecina
  ‘the neighbor’

This asymmetry would suggest that an English determiner cannot appear together with a Spanish noun. As Liceras et al. (2008) make clear, such an asymmetry is not factually attested. Examples like (48) are attested in spontaneous production (see also Liceras et al., 2005, 2008; Pierantozzi, 2012; López, 2018). Liceras et al. (2005, 2008) also show that the Spanish determiner is preferred in language use. The scholars suggest that such a preference can be accounted for by what they label the Grammatical Features Spell-out Hypothesis (GFSH). The GFSH holds that functional categories containing highly ‘grammaticized’ features will be chosen. Because they have gender, Spanish determiners contain more features than English determiners, and therefore Spanish determiners will be preferred. As Grimstad et al. (2018) point out in their discussion, the GFSH is a hypothesis about production preferences which is guided by a grammatical mechanism on the PF side.

Now, scholars do not always have large corpora available to make the comparison that was just made. However, such findings as in the English-Spanish mixing case may caution us to draw too big conclusions based on acceptability data alone. Judgments involving mixing are often negative due to sociolinguistic reasons, suggesting that they often should be combined with corpus evidence when such evidence is available (see e.g., González-Vilbazo et al., 2013 for relevant discussion).

However, assuming that the data reported are adequate, a further option would be to appeal to morpho-phonology in accounting for why some data points do not fit the overall generalization. That is, in the spirit of MacSwan (1999, 2000, 2005, 2013), Bandi-Rao and den Dikken (2014), and MacSwan and Colina (2014) and research cited there, those cases where a root and functional morphology are not able to combine must be ill-formed due to some PF-rule. This would be a language specific rule that would hopefully relate to other properties in the grammar, e.g., as to whether or not a specific functional element is able to incorporate with a root (cf. Bandi-Rao and den Dikken, 2014).

Approaching this problem from the perspective of Distributed Morphology (Embick and Noyer, 2007; Embick, 2015; Alexiadou, 2017a,b), we assume that nouns and verbs are syntactically derived. In particular, they emerge when a-categorial roots combine with categorizing heads (v and n):

**Table:** 

(49)	a.	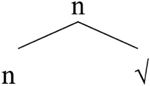	b.	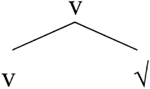

As already mentioned, we further assume that information about inflectional class, gender, and case is realized on n, for nouns (e.g., Alexiadou, 2004, 2017b, Kramer, 2015), and v hosts verbalizers across languages. Once categorized, nP and vPs become part of extend projections, which we assume to be identical across languages. When it comes to bilingual speakers it is important to distinguish between utterances in monolingual mode and those in bilingual mode. Assuming that the abstract clausal structure is universal, these productions will differ in terms of realization and flavors of heads present in the structure (see Grimstad, 2017 for extensive discussion of this point). Speakers are able to shift from mode to mode, suggesting that in the monolingual mode alternative realizations are blocked. In the bilingual mode, matters are more complex. Let us illustrate this by discussing two of our patterns:

**Table:** 

(50)	Spanish root + German affix	vs.	^∗^German root + Spanish affix
(51)	German/English root + Greek affix	vs.	^∗^Greek root + German/ English affix

Both patterns involve cases where a root in combination with v or n create the vP and nP phase, respectively (cf. Marantz, 2001, 2007 and Arad, 2003, 2005). In both cases, the complement of the phase head comes from one language, while the realization of n, v from the other language. We have rejected above the GFSH, which appeals to visibility, though at first sight our data seem compatible with this, as in (50) and (51), the realization of v an n seems to come from the language that makes more distinctions within a domain (e.g., case, gender, declension classes, conjugation classes, etc.). But note that it is not the case that all possible realizations of v and n are found in the data. This is particularly clear in the Greek case of mixing in the verbal domain, where the default verbalizer -*ar*- is used, although the language has a very rich system of verbalizers. Similar observations can be made for the nominal domain, where mixing does not distribute nouns equally across the 8 declension classes of Greek but instead picks class 2 for masculine, class 3 for feminine, and class 6 for neuter nouns [assuming Ralli (1994) classification, see also Alexiadou and Müller (2008)]. Thus, it seems to us there is a default mechanism to integrate Germanic roots into Greek morphology: speakers pick the default/underspecified realization, if such a realization is available. That is, the bilingual speaker in view of the fact that she has more VIs at her disposal will pick an overt realization, if a default such realization is available. The default realization is the one that is compatible with the largest number of roots, i.e., the roots of both languages. This competition is determined on the basis of the available VIs for the individual language pairs: e.g., -*ar*- is the default realization of v in the case of Greek and Germanic pairs, while -*isier*- the default realization of v in the case of Spanish and German pairs, as Spanish has no overt realization of v.

Let us illustrate this idea in some detail. We begin with the observation that in the nominal domain, as also stated in López (2018), two options are available to speakers: either to make use of the default marking in, e.g., Spanish (masculine), Greek (masculine for animates, neuter for in-animates) or to associate with the gender of the translation equivalent, i.e., adopt the analogical transfer strategy. By contrast, it is not clear what the default gender is in German, for reasons that have to do with gender shift in the history of the language (neuter to masculine; Steinmetz, 1997). Thus, in the case of the Spanish-German pairs, the system treats masculine and neuter alike, while feminine nouns are always marked feminine. In the Greek mixing pairs, the blocking of Germanic affixes on Greek roots is probably a PF effect, as we will show below for the verbal domain as well. For instance, a Greek root cannot appear ending in a consonant, thus bearing zero (German or English) morphology. A German/English root can, however, and this is why (17) is also possible. Support for our view on how VIs are chosen comes from another set of data discussed in López (2018) involving Swahili/English word-internal mixing: English nouns are used in such contexts, and they always have a Swahili noun class prefix. As noun classes play a role similar to gender and are associated with n, this pair behaves similar to the other varieties we have been discussing here.

Matters are different in the verbal domain. We hold that -*ify*- and its cognates across languages, i.e., Greek -*ar*-, German -*isiere*- are realizations of v. In languages where v is overtly realized by these forms, which are the default ones, roots combine with these to form the word-internal mixed cases, just as we have seen in the data reviewed in this paper.

Spanish lacks verbalizers, although it has verbal conjugations. We assume that the features related to conjugation are attached post-syntactically (Oltra-Massuet, 1999). Nevertheless, the features need to match the root in order for the appropriate conjugation to appear, ruling out Spanish inflection with German roots, again a PF effect.

Alexiadou (2017a, p. 183) provides additional examples here given in (52), which are similar to the data in (30) showing that German roots cannot combine with Spanish inflection.

(52) a. ^∗^**kämpf**-ar-o
  fight-AFF-1SG
  b. ^∗^s**chwim**-ar-o
  swim-AFF-1SG
  c. ^∗^**lauf**-ar-o
  run-AFF-1SG

These examples show that a Greek verbalizer cannot combine with a German root. Alexiadou (2017a, p. 184) argues that these examples are ruled out for morphophonological reasons. She points out that word-internal and word-initial consonant clusters are dis-preferred in Greek. For that reason, Greek speakers instead make use of the light verb strategy, as seen in (5a), repeated here as (53).

(53) kano **abschalten**
  do.1SG kick.back.INF
  ‘I am kicking back.’

Furthermore, it should be noted that the examples in (52) contain either an umlaut or a diphthong. Neither of these exist in Greek phonology. Since Greek supplies the v, the output of word formation via incorporation needs to adhere to Greek phonotactics.

Considering English-Norwegian, speakers combine English roots with Norwegian functional morphology, they generally do not combine Norwegian roots with English functional morphology. This is arguably because they are in a ‘Norwegian’ language mode. However, as Grimstad (2017) shows, they do use English morphology in the verbal domain, though importantly, only in combination with English roots. In the nominal domain, there are cases of English functional morphology appearing with English roots (see Haugen, 1953 and in particular Riksem, 2017). We do not find cases of Norwegian roots appearing with English functional morphology, which may be due to Norwegian being a heritage language and therefore, when English functional morphology is used, speakers will not insert a Norwegian root (as we know that they have quite significant problems with lexical access, see again Grimstad, 2017, 2018 and Riksem, 2018).

Telugu is a bit more complex basically because *inc* is not exactly identical to *ify*, as it can be used to form non-lexical causatives as well. This means that it might very well be that *inc* realizes something higher than our verbalizing v, i.e., it is the realization of a *make* type v head, which takes a vP as its complement. This would explain why it would not be able to merge with an English root: Assuming that the combination between the verbalizer and the root is local, *inc* might simply be a realization of a v head in a higher phase, and thus it cannot combine with the English root. Moreover, note that the ungrammatical examples cited in Bandi-Rao and den Dikken (2014) involve a stative verb combining with *inc*. That might very well be accidental. If not, however, and if *inc* is more like English *do/make*, we may get a different flavors of v effect, meaning that there may be incompatibility between stative psych roots and causative/eventive semantics. A more in-depth investigation of Telugu would be required in order to investigate this, which goes beyond the scope of the current paper.

The fact that speakers pick overt default realizations seems to suggest that all illicit combinations are filtered-out at PF. We assume as in Embick (2010) that the phase head determines also the phonology of the whole phase, see also López et al. (2017) for further evidence. We mentioned earlier that several word-internal switches in Greek are filtered out because of phonotactics (cf. MacSwan, 1999, 2000; MacSwan and Colina, 2014). Therefore, it seems plausible to assume that if speakers can pick among different types of n/v to combine with roots, they pick those that will fit the general phonology/properties of the phase head. Put differently, the phonology within a phase head needs to be uniform. This is a far more refined role of phonology than an across-the-board ban on word-internal mixing.

Finally, note that what we discuss here is largely compatible with López’s (2018) view and model. There are, however, several issues and questions that we would like to raise. A first issue relates to the problem of root-equivalence, i.e., the question of whether roots from two different languages are interpreted identically by the Encyclopedia, List 3 in Distributed Morphology. We agree with López (2018) that most likely this is rarely the case (see also Grimstad, 2018 and Riksem, 2018 for discussion). Does this suggest that the two forms have very different contexts of use or is it simply an issue of retrieval? Moreover, it is not the case that languages have the same inventory of roots (Alexiadou and Lohndal, 2017a), and the implications of this should be examined in the context of language mixing.

In addition, we think that his system predicts a lot more of free variation than it is actually found in the data, a point López (2018) himself also acknowledges. Moreover, the system predicts the possibility of double realization of a particular feature. Though such cases do exist, they are certainly limited. Finally, it is not entirely clear how the competition between different realizations of a particular feature is resolved. In other words, assuming the subset principle (Halle, 1997), how do we decide which form is more specific, the L1 or the L2 one? López (2018) argues that the competition does not take place, as the conditions for insertion of vocabulary items are very different. We have outlined above a system that favors overt realizations but picks default forms, thus blocking double realization.

## Conclusion

In this paper, we have surveyed instances of word-internal language mixing across several different language pairs. In general, a root from one language can combine with functional morphology from another. In cases where such a combination is not licit, we have argued that there may be two reasons why this is the case: Either because the language mode of the speaker suggests that the functional morphology should come from the language with overt default realization or because morpho-phonological reasons rule out the particular mixing in question. We have also shown how a decompositional model like Distributed Morphology can be utilized to analyze the patterns.

## Author Contributions

All authors listed have made a substantial, direct and intellectual contribution to the work, and approved it for publication.

## Conflict of Interest Statement

The authors declare that the research was conducted in the absence of any commercial or financial relationships that could be construed as a potential conflict of interest.

## Footnotes

1 The literature is ripe with discussions of how to define words, an issue we won’t delve further into. See, among many, Katamba (2004), Katamba and Stonham (2006), and Anderson (2015) for good discussions.

2 Other scholars do not agree, arguing that speakers can distinguish between nonce borrowing, established loans, and (multiword) code switches (Poplack and Dion, 2012; Poplack, 2018). In particular, Poplack (2018, pp. 156–157) argues that differential patterns of language use related to these three categories demonstrate their psychological reality. We do not disagree that they may behave differently, but the question is whether or not this reflects the underlying linguistic competence of the speaker, at the fine-grained level of minimal syntactic units. The current contribution suggests that they may not differ.

3 Grimstad et al. (2014) provide an analysis of how these bigger mixed chunks can be analyzed within this framework (see also Grimstad, 2017). Another similar type of exception involves the use of the English plural marker -*s* with an English noun in an otherwise Norwegian noun phrase. See Riksem (2018) on this phenomenon.
